# Parkinson Disease Signaling Pathways, Molecular Mechanisms, and Potential Therapeutic Strategies: A Comprehensive Review

**DOI:** 10.3390/ijms26136416

**Published:** 2025-07-03

**Authors:** Muhammad S. Khan, Somayyeh Nasiripour, Jean C. Bopassa

**Affiliations:** Department of Cellular and Integrative Physiology, School of Medicine, University of Texas Health Science Center at San Antonio (UTHSCSA), 7703 Floyd Curl Dr., San Antonio, TX 78229, USA; sohail.bannu@gmail.com (M.S.K.); nasiripour@uthscsa.edu (S.N.)

**Keywords:** Parkinson’s disease (PD), oxidative stress, ferroptosis, mitochondrial dysfunction, neuroinflammation, gut dysbiosis, medicinal plants

## Abstract

Parkinson’s disease (PD) is considered the second most common neurodegenerative disease worldwide; treating this disease remains quite challenging. Environmental and genetic factors may play a role in the pathophysiology of PD. α-synuclein aggregation, oxidative stress, ferroptosis, mitochondrial failure, neuroinflammation, and gut dysbiosis are among the known risk factors of PD. The pathophysiology of Parkinson’s disease is complicated by the interconnections between these molecular pathways, which also present significant obstacles to treatment development. However, due to its complex mechanism and long latency, PD is difficult to diagnose and detect, which presents a barrier to treatment. In addition, the need to develop new treatments for PD is increased by the fact that the majority of traditional therapeutic methods have major side effects and limited effects. Therefore, a deeper understanding of the fundamental mechanisms underlying PD is required. This review provides a comprehensive analysis of the current landscape of PD pathophysiology, paying particular attention to the molecular processes of PD, as well as the traditional research models, clinical diagnostic standards, documented medication therapeutic approaches, and recently disclosed drug candidates in clinical trials. We also highlighted the herbal-derived components that have recently been identified for their effects in the treatment of PD to provide a review and perspectives for the development of the next generation of drugs and preparations for the treatment of PD.

## 1. Introduction

**Figure 1:**
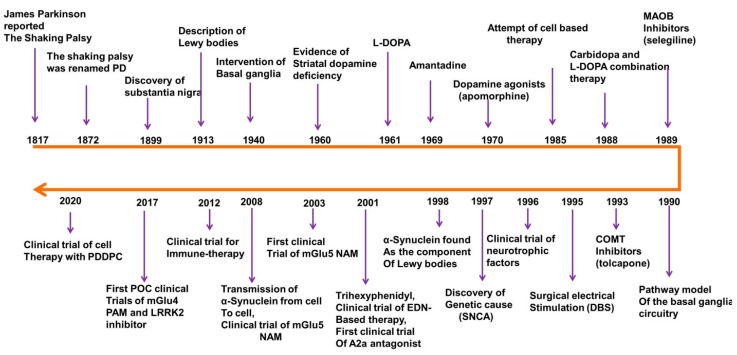
Basic research and drug development history for PD disease and therapy, modified from Charvin et al., 2018 [5]. A2a adenosine receptor subtype 2a, mGlu metabotropic glutamate receptor, NAM negative allosteric modulator, PAM positive allosteric modulator, EDN embryonic dopamine neuron, PDDPC personalized iPSC-derived dopamine progenitor cell, iPSC induced pluripotent stem cell, DBS deep brain stimulation. This figure was generated by using Microsoft PowerPoint.

Parkinson’s disease (PD) is the second most prevalent neurological illness worldwide, with its prevalence increasing by 74.3% between 1990 and 2016. James Parkinson was the first to describe Parkinson’s disease (PD) as a neurological condition in his 1817 monograph, “an Essay on the Shaking Palsy”. A succession of researchers, starting with Jean-Martin Charcot, has contributed to a thorough explanation of the clinical spectrum and anatomical foundation of PD, including neuropathological alterations of the substantia nigra (SN), Lewy bodies, motor and non-motor symptoms, and dopamine (DA) function. Following these discoveries (Figure 1), extremely effective treatments emerged to successfully manage the symptoms, such as deep brain stimulation and pharmaceutical DA substitution (levodopa therapy). However, none of these therapies can reverse the progression of PD, especially the worsening of treatment-resistant motor and non-motor symptoms that continue to plague the difficult lives of Parkinson’s patients. To provide a summary and outlook for the development of the next class of drugs and therapies for the treatment of PD, we summarized the molecular principles underlying PD and the research models, characterized the clinical features and diagnostic criteria of PD, listed drugs used in clinical practice and on the market, and discussed the available treatments for PD like such as compounds [1,2,3,4].

## 2. Epidemiology, Clinical Features, and Diagnostic Criteria

After age 60, the prevalence of PD increases sharply to over 3% among people aged 80. Men are more likely than women to have Parkinson’s disease in most populations. The disparity in frequency between locations and races is probably explained by differences in living conditions and environment. Although dietary practices can modify the prevalence of the disease, environmental pollutants can cause symptoms of PD. Notable examples include reduced risks among smokers and frequent caffeine users. The majority of clinical features of PD are composed of both motor and non-motor symptoms. A range of motor symptoms, including bradykinesia, muscle stiffness, rest tremor, and posture and walking problems, are present in patients with PD. Based on clinical data, PD can be classified into two basic types: tremor-dominant PD and non-tremor-dominant PD. Tremor-dominant PD is often associated with less functional impairment and a slower rate of development than non-tremor-dominant PD [6,7,8,9,10,11,12,13,14,15,16,17]. Constipation, melancholia, olfactory dysfunction, cognitive impairment, and sleep problems include excessive daytime sleepiness and fast eye movement. Non-motor features include pain, fatigue, autonomic dysfunction, and sleep behavior disorders. In the early stages of PD, non-motor symptoms often occur before motor symptoms. Some problems, including psychosis, motor and non-motor fluctuations, dyskinesia, decline in motor functions, and long-term symptomatic medication, will occur as PD progresses. Up to 50% of patients with PD report choking, and up to 80% of patients experience gait freezing and falls after about 17 years of the disease. It is also thought that people who have had PD for at least 20 years are more likely to develop dementia. One of the main pathogenic features of PD is the progressive loss of only a portion of neurons in specific brain regions, such as the SN. In early stages, dopaminergic neurons are lost only in the ventrolateral SN, but, in later stages, this damage spreads.

Additionally, abnormally high amounts of α-synuclein collect in the cytoplasm of some neurons in various parts of the brain. Lewy bodies, a common sign of neuropathology, are produced by aggregated α-synuclein in olfactory neurons, brainstem cholinergic and monoaminergic neurons, and other neurons. As PD progresses, Lewy bodies proliferate and affect not only the limbic and neocortical areas of the brain but also non-dopaminergic neurons in other parts of the brain [18,19,20,21,22,23,24,25,26,27]. Finally, PD causes the degeneration of neurons outside the central nervous system (CNS), such as those in the mesenteric or olfactory bulb. Due to the complexity of disease presentation and the risk of early symptoms of PD, 10% of diagnosed patients were initially classified as having other diseases. The International Parkinson and Movement Disorder Society has developed criteria to improve diagnostic accuracy. These guidelines state that Parkinson’s syndrome is defined as the presence of bradykinesia plus at least one additional cardinal motor sign (4–6-Hz rest tremor or limb rigidity). “Red flags” for alternative diagnoses and excluded clinical features were also mentioned. Clinical diagnosis of PD often relies on genetic testing, diffusion-weighted magnetic resonance imaging (MR-DWI), structural magnetic resonance imaging (MRI), and DA transporter–single-photon emission computed tomography (DATSPECT). Because approximately 90% of people with PD experience hyposmia or anosmia, testing of olfactory function using the UPSIT or Sniffin Stick tests is sometimes part of the initial clinical examination. Advanced magnetic resonance imaging techniques have revealed MRI features that are highly specific for atypical Parkinsonism. These include neuromelanin imaging (NMI), which utilizes the paramagnetic properties of neuromelanin, and quantitative susceptibility mapping (QSM), which determines iron accumulation in the SN. Interestingly, NMI may indicate changes in prodromal PD. Ioflupane I-123 single-photon emission computed tomography (SPECT) is another method used to differentiate PD from clinical symptoms that are not associated with presynaptic nigrostriatal terminal dysfunction [28,29,30,31,32,33,34,35,36]. Despite these advances, there is still room for improvement in one area of clinical diagnosis: the application of genetic testing, which is now only used when a specific hereditary etiology is suspected. But as PD progresses, we also see an increase in the number of genes associated with complex symptoms, including Parkinson’s symptoms. Regular genetic testing may be beneficial in certain situations.

## 3. Etiology and Pathogenesis of PD

### 3.1. Genetic and Environmental Factors and Pathogenesis of PD

Both hereditary and environmental variables play a role in the complex etiology of PD. Environmental factors include things like stress, brain damage, physical inactivity, and pesticide exposure. The symptoms of 1-methyl-4-phenyl-1,2,3,6-tetrahydrodropyridine (MPTP) poisoning are almost the same as those of PD, and the main metabolite of MPTP, 1-methyl-4-phenylpyridinium (MPP+), shares structural similarities with paraquat. According to human epidemiological research, living in a rural area and being exposed to pesticides and herbicides are linked to an increased risk of PD [37,38,39,40,41]. However, convincing evidence linking certain chemicals to PD is still required.

### 3.2. Autosomal-Dominant Genes and PD Pathogenesis

The *PARK1*/*PARK4* gene for α-synuclein expression has been shown to be linked to aberrant pathological aggregation of insoluble α-synuclein fibril in patients with SNCA mutations whose brains exhibited α-synuclein aggregation, manifesting as DA neuron loss and the appearance of Lewy bodies. A mutation in the leucine-rich repeat kinase 2 (*LRRK2*) gene, also known as PARK8, is the most common genetic cause of Parkinson’s disease. The majority of people with late-onset LRRK2 mutations are over 50 years old. G2019S, R1441C, R1441G, and R1441H are the most prevalent variants of this mutation, which can disrupt a number of physiological functions, such as vesicle transport, cytoskeletal function, protein synthesis, and the lysosomal system, leading to the death and degeneration of DA neurons. PARK13, the HTRA2 serine peptidase 2 (*HTRA2*) gene, has been shown to be essential for preservation of regular mitochondrial function and is released into the cytoplasm from damaged mitochondria. Under stressful circumstances, it has also been shown to have a neuroprotective effect; PARK13 mutant animals exhibit increased reactive oxygen species (ROS), mitochondrial dysfunction, and PD symptoms [42,43,44,45,46,47]. PINK1-mediated phosphorylation regulates its activity, which is essential for preserving mitochondrial integrity under stressful situations. Degradation of misfolded SNCA is central to PARK13. Endosome proteins from the pre-lysosomal compartment network must be retro-transferred to the trans-Golgi network via the PARK17 gene, which is a component of the reverse transcriptome complex (VPS35). In an endosomal compartment, the cation-independent mannose 6-phosphate receptor (CI-MPR) may attach to VPS35 and become trapped in recycling tubules, blocking its transfer to lysosomes or vacuoles. The PARK18 gene, which encodes the eukaryotic translation initiation factor 4 gamma 1 (EIF4G1), regulates the start of the translation of mRNAs encoding growth, survival, and mitochondrial proteins in response to various stimuli. Two mutations, EIF4G1 p.A502V and EIF4G1 p.R1205H, have been shown to alter the eIF4G1-eIF4E or eIF4G1-eIF3e binding, which is thought to act as the molecular bridge between the mRNA cap-binding complex and the 40S subunit and cause mitochondria-related imbalance [48,49,50,51,52,53,54,55,56].

### 3.3. Autosomal-Recessive Genes and PD Pathogenesis

The parkin RBR E3 ubiquitin protein ligase gene (PRKN) encodes PARK2, the most common cause of autosomal-recessive early-onset Parkinson’s syndrome. Studies show that up to 50% of PD cases above the age of 25 and up to 7% of patients between the ages of 30 and 35 had the PARK2 mutation. Parkin is an E3 ubiquitin ligase that functions as an E1 ubiquitin-activating enzyme and an E2 ubiquitin-conjugating enzyme to break down specific proteins in the ubiquitin–proteasome system, which is believed to be a multifunctional neuroprotective agent against a range of toxic injuries, including mitochondrial poisons, and is crucial for the survival of DA neurons. The PTEN-induced putative kinase 1 gene (PINK1) encodes the serine/threonine protein kinase PARK6, which has been shown to interact with parkin to promote selective autophagy in depolarized mitochondria and preserve mitochondrial integrity. After being phosphorylated by PINK1 to activate its E3 ligase activity, parkin is normally recruited to depolarized mitochondria to initiate autophagy and remove the damaged or defective mitochondria. Because it cannot be sent to the inner mitochondrial membrane for breakdown, PINK1 accumulates in the outer mitochondrial membrane of inefficient mitochondria to initiate the removal of damaged mitochondria from the cell. The parkinsonism-associated deglycase gene PARK7, often referred to as DJ-1, protects DA neurons in the model system against damage caused by mutant synuclein, rotenone, 6-hydroxydopamine (6-OHDA), and hydrogen peroxide. PARK9, the ATPase 13A2 gene (ATP13A2), encodes transmembrane endo-/lysosomal related proteins, and the lysosomal signaling lipids phosphatidylic acid and phosphatidylinositol (3,5) biphosphate interact with the N-terminus of ATP13A2, which acts as the catalyst for ATP13A2 action and regulates endo/lysosomal cargo sorting. Most ATP13A2 mutations affect its functional domains, specifically, its transmembrane and E1-E2 adenosine triphosphatase domains. If ATP13A2 is functionally lost, this may result in Zn^2+^ dysregulation and abnormal cell metabolism, including dysfunctional energy production and decreased lysosomal proteolysis [57,58,59,60,61,62,63,64,65,66,67,68]. Furthermore, it has been shown that ATP13A2 lessens α-synuclein’s neurotoxicity. The F-box protein 7 gene (FBXO7) encodes the PARK15 protein, which is a subunit of the F-box protein that acts as an adapter protein in the SKP1/cullin-1/F-box protein E3 ubiquitin ligase complex. It recognizes and mediates the non-degrading ubiquitination of the translocase of outer mitochondrial membrane 20 and glycogen synthase kinase (GSK-3β). This process regulates mitophagy, mitochondrial motility, mitochondrial membrane potential, mitochondrial bioenergetics, biogenesis, and apoptosis linked to the mitochondria. A malfunction may also reduce complex-I’s activity in the electron transport chain, which would lower ATP levels and mitochondrial membrane potential while increasing cytoplasmic ROS because FBXO7 is stress-responsive [69,70,71,72,73,74,75].

## 4. Molecular Mechanisms of PD

### 4.1. Role of α-Synuclein Aggregation in PD Pathology

Numerous molecular and cellular alterations, such as α-synuclein aggregation, abnormal protein handling, excitotoxicity, oxidative stress, apoptosis, and mitochondrial dysfunction, have been connected to neural degeneration. One of the most significant theories explaining the loss of nigrostriatal neurones in Parkinson’s disease is abnormal α-synuclein aggregation. A putative chaperone, α-synuclein, is found in the cytosol, mitochondria, and nucleus and is involved in intracellular trafficking, synaptic vesicle dynamics, and mitochondrial activity. According to some data, the protein may be involved in the brain’s lipid metabolism, which has a role in the pathophysiology of Parkinson’s disease. When soluble α-synuclein monomers combine to form oligomers, which then form small protofibrils and finally giant, insoluble fibrils, α-synuclein itself can become neurotoxic. The accumulation of α-synuclein may be significantly influenced by age-related declines in the brain’s proteolytic defense mechanisms [76,77,78,79,80,81,82,83,84]. In particular, the lysosomal autophagy and ubiquitin–proteasome mechanisms preserve intracellular α-synuclein homeostasis. α-synuclein cleavage is sometimes attributed to extracellular proteases that are not a component of either system. Thus, α-synuclein buildup may be facilitated by disruption of these degradation processes.

### 4.2. Role of Oxidative Stress (OS) in PD Pathology

**Figure 2:**
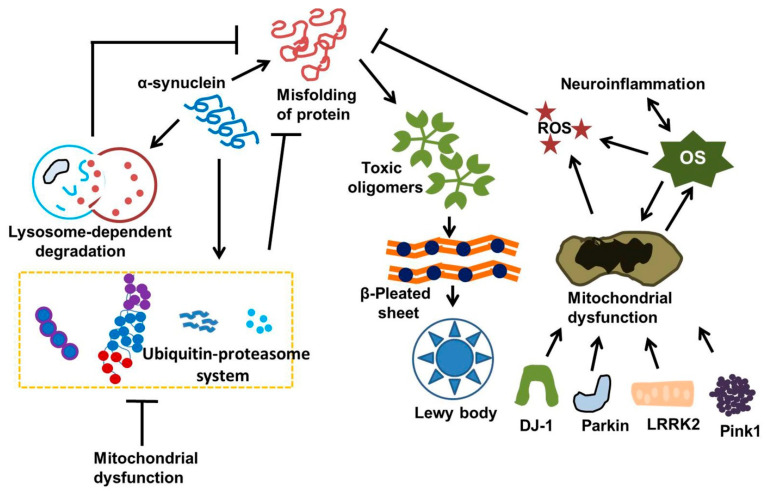
Intracellular α-synuclein balance is preserved through the ubiquitin–proteasome and lysosomal autophagy pathways. Dysfunction of these degradation systems due to oxidative stress, mitochondrial issues, or neuroinflammation may lead to the buildup of α-synuclein. Additionally, gene mutations such as LRRK2, DJ-1, Parkin, and Pink1 lead to mitochondrial impairment and enhance cell mortality. Ultimately, OS and neuroinflammation seem to be linked. This figure was generated by using Microsoft PowerPoint. This figure was modified from Xu Dong-Chen et al., 2023 [103].

One of the main aging processes that directly damage the central nervous system is oxidative stress (OS). Free radicals, or ROS, play a crucial role in apoptosis, gene transcription, host defense, and the regulation of neural plasticity under physiological settings. On the other hand, OS happens when ROS outweighs antioxidant action in cells. In different neurons, including DA-neuronal tissue, cytotoxic chemicals then build up to induce cell death, lipid breakdown, protein collapse, and enzyme failure (Figure 2).

These dysfunctions may be the cause of Alzheimer’s disease (AD), as well as contribute to the pathophysiology of Parkinson’s disease (PD). At the moment, NADPH oxidase (NOX) is thought to be the most significant ROS generator, and it is essential for initiating OS and neurotoxicity. ROS is also produced in large quantities by mitochondria. The majority of ROS is believed to be generated in mitochondria by complexes I and III of the electron transport chain. One electron moving from one oxygen molecule to another oxygen molecule produces the superoxide radical, the primary ROS produced in mitochondria. The superoxide radical may be converted to hydrogen peroxide by superoxide dismutase 2 (MnSOD), which can then be detoxified by the catalase enzyme. However, when metal ions like Fe^2+^ are present, hydrogen peroxide can change into a highly reactive hydroxyl radical due to the Fenton reaction, which severely oxidizes DNA or lipids. Iron ion homeostasis imbalance is linked to the mechanism of ferroptosis, suggesting a link between ferroptosis and OS. The hydroxyl radicals from the Fenton reaction can oxidize lipids to create lipid peroxides, which can lead to ferroptotic cell death [85,86,87,88,89,90,91,92,93,94,95,96,97,98]. Another biochemical indicator of ferroptosis is glutathione depletion, which exacerbates intracellular OS by encouraging the accumulation of lipid peroxides that cause ferroptosis. Moreover, elevated OS can damage the lysosomal autophagy mechanism and reduce lysosomes, linking OS to the accumulation of α-synuclein. According to a different theory, DA-quinones can be produced by simply oxidizing excess cytosolic DA. Then, α-synuclein may self-assemble as a result of the DA quinine-modified α-synuclein partially inhibiting chaperone-mediated autophagy. In the meantime, intracellular α-synuclein aggregation development raised mitochondrial OS [99,100,101,102].

### 4.3. Role of Ferroptosis in PD Pathology

**Figure 3:**
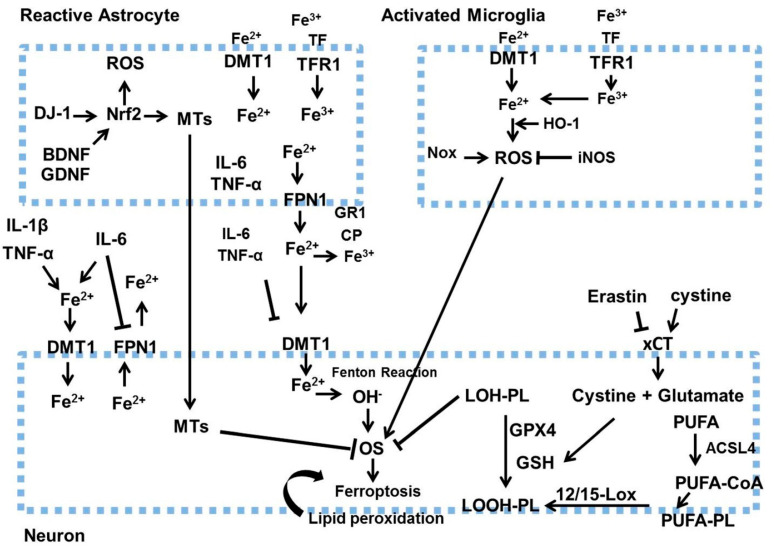
Initially, inflammatory cytokines (IL-1β, TNF-α, IL-6) secreted by activated microglia and astrocytes enhance iron build-up in neurons by increasing DMT1 expression and decreasing FPN1 expression. Activated astrocytes release BDNF and GDNF, which decrease iron buildup in neurons by lowering DMT1 levels. Secondly, ROS generated by activated microglia enhances neuronal oxidative stress. The increase in Nrf2 and the secretion of metallothioneins in astrocytes enhance neuronal resistance to oxidative stress. BDNF stands for brain-derived neurotrophic factor, GDNF refers to glial cell line-derived neurotrophic factor, HO-1 is heme oxygenase-1, IL-1β denotes interleukin-1β, IL-6 signifies interleukin 6, iNOS indicates inducible nitric oxide synthase, NOX represents NADPH oxidase, Nrf2 is nuclear factor-erythroid factor-2, Tf stands for transferrin, TNF-α is tumor necrosis factor α, 12/15-LOX refers to lipoxygenases 12/15, LOOH-PL means lipid hydroperoxide–phospholipid, and LOH-PL signifies lipid alcohol–phospholipid. This figure was generated by using Microsoft PowerPoint. This figure was modified from Xu Dong-Chen et al., 2023 [103].

Ferroptosis, an iron-dependent form of regulated cell death that leads to OS and cell death, is brought on by an abnormal iron metabolism and extreme lipid peroxidation (Figure 3).

It was also linked to the loss of DA neurons in Parkinson’s disease. In the cytosol, coenzyme A (CoA) is changed into free polyunsaturated fatty acids (PUFAs) by the enzyme acyl-CoA synthetase long-chain family member 4 (ACSL4). Lipid peroxidation can then result from the incorporation of PUFA-CoA into phospholipids, which are oxidized by lipoxygenases 12/15. Glutathione (GSH), an antioxidant produced by the body from glutamate and cysteine, can decrease ferroptosis by stopping lipid peroxidation. Cysteine, the rate-limiting substrate, can be absorbed by the xCT antiporter as an oxidized cysteine dimer or generated from methionine by the transsulfuration pathway. DJ-1, another cellular antioxidant enzyme, acts as a ferroptosis inhibitor and protects the synthesis of cysteine and GSH by preventing the destruction of the transsulfuration pathway [104,105,106,107,108]. Glutathione peroxidase 4 (GPX4) is the sole member of the glutathione peroxidase family that can reduce lipid hydroperoxides in physiological conditions. GPX4 needs reduced GSH to convert lipid hydroperoxides to lipid alcohols, and direct inactivation of GPX4 by RAS-selective lethal is one of the most widely used experimental techniques to cause ferroptosis. Ferroptosis genes themselves can be linked to Parkinson’s disease (PD), and the characteristics of ferroptosis induction are extremely comparable with the pathogenic changes observed in PD patients. Iron and α-synuclein coexist in Lewy bodies in the midbrain of Parkinson’s disease patients. As a metal-binding protein, α-synuclein will alter its conformation when it binds iron, which will cause it to aggregate [109,110,111,112,113,114,115]. Astrocytes and microglia control iron homeostasis in brain networks. Iron deposition in the central nervous system may increase as a result of iron buildup in activated microglia and the consequent generation of proinflammatory cytokines. Neuronal iron deposition was exacerbated by ferroportin 1 (FPN1), which drastically downregulated expression, while divalent metal transporter 1 (DMT1), iron regulatory protein 1, and transferrin receptor 1 (TfR1) significantly increased expression. NOX in active microglia not only produced ROS but also resulted in OS, which triggered ferroptosis in DA neurons. Additionally, microglia responded to inflammatory signals by significantly increasing inducible nitric oxide synthase (iNOS), which enabled it to suppress 15-lipoxygenase activity and prevent ferroptosis. Astrocytes primarily use protein interaction cascades, especially ceruloplasmin (CP), to transport different types of iron. Iron efflux from cells may be facilitated by the effective oxidation of Fe^2+^ into Fe^3+^ by CP’s ferroxidase activity, but nearly 80% of this activity was diminished in PD patients’ SN, indicating that lower CP expression and the resulting iron accumulation contribute to neuronal death in PD. This overexpression of heme oxygenase-1 by microglia and astrocytes will also contribute to the neurotoxic accumulation of iron in the brain. Reactive astrocytes may also generate other antioxidant molecules, like GSH and metallothioneins, to eliminate ROS from DA neurons. In order to shield the DA neurons from OS, Nrf2 activation in astrocytes can also increase antioxidant enzymes, such as MTs and GSH synthesis enzymes [112,113,114,115,116,117,118,119,120,121,122,123,124,125,126,127].

### 4.4. Role of Mitochondrial Dysfunction in PD Pathology

The significance of mitochondrial dysfunction in the pathophysiology of Parkinson’s disease is increasingly acknowledged. Several studies have found that ongoing ROS generation and dopaminergic neurotoxicity result from mitochondrial dysfunction. Observations after the targeted inhibition of mitochondrial complex I through MPTP infusions offered the initial proof of this connection. Additional inhibitors of this complex, including rotenone, pyridaben, fenpyroximate, and trichloroethylene, exhibited similar negative effects. Additionally, animals with elevated levels of α-synuclein were more susceptible to toxins compared to mice without it, suggesting that mitochondrial α-synuclein enhances toxicity. Key contributors to mitochondrial dysfunction are believed to be the dysregulation of transcription factors and the subsequent changes in mitochondrial biogenesis. A key regulator of mitochondrial biogenesis is the transcription-factor coactivator peroxisome proliferator-activated receptor gamma coactivator-1a (PGC-1a). Although PGC-1a overexpression protects against neurotoxicity, dopaminergic cells in PGC-1a-deficient mice exhibit increased susceptibility to MPTP. Furthermore, harmful mutations in certain genes, such as Parkin, DJ-1, LRRK2, and PINK1, may lead to mitochondrial dysfunction. E3, a ligase of ubiquitin proteases, is produced by Parkin. Animals lacking Parkin are especially susceptible to the mitochondrial complex-I inhibitor known as rotenone. Mutations in PINK1 lead to an autosomal-recessive form of Parkinson’s disease (PD), probably through enhancing α-synuclein aggregation and reducing mitochondrial respiration and ATP production [128,129,130,131,132,133,134,135,136,137,138,139,140]. Additionally, it seems that PINK1 failure affects mitophagy and results in abnormalities in mitochondrial localization. In Drosophila, studies on combined Parkin and PINK1 knockouts revealed that they are part of the same pathway, with PINK1 upstream of Parkin. Parkin is recruited by the cytosol to mediate selective autophagic clearance of damaged and depolarized mitochondria. Dysfunctional mitochondria must collect PINK1 and activate kinases in order for Parkin to translocate. Given that SHP2 knockdown prevents the process, research indicates that Src homology 2 domain-containing tyrosine phosphatase-2 (SHP2) is crucial for mitochondrial translocation and ubiquitination of Parkin. One possible method by which SHP2 regulates Parkin activity is tyrosine dephosphorylation. Lovastatin is a medication that may be used to treat Parkinson’s disease because it increases SHP2 activity. In addition to increasing vulnerability to OS-induced cell death, loss-of-function mutations in the DJ-1 gene also result in a rare autosomal-recessive type of Parkinson’s disease. Humans with DJ-1 mutations and DJ-1 knockout animals both have mitochondria with compromised respiration [141,142,143,144,145,146,147,148,149,150]. On the other hand, LRRK2 mutations are linked to autosomal-dominant Parkinson’s disease. In Caenorhabditis elegans with G2019SLRRK2 mutations, the DA neurons showed mitochondrial abnormalities, as did the striatum of older homozygous LRRK2G2019S knock-in mice. Generally speaking, LRRK2 mutations mediated by dynamin-like proteins are linked to mitochondrial fission [151,152,153].

### 4.5. Role of Neuroinflammation in PD Pathology

Preclinical and clinical research has shown that neuroinflammation may influence the pathophysiology of PD, linked to its initiation and advancement. Analysis of the brains of Parkinson’s disease (PD) patients after death has shown signs of damage associated with neuroinflammation through both cellular and molecular investigations. The progression of PD is shaped by both inherent and adaptive immune responses. Activated microglia, innate immune cells residing in the brain, elevate nuclear factor kappa-B (NF-κB) and NLR family pyrin domain-containing 3 (NLRP3), leading to an increase in cytokines such as TNF-α and IL-1β. Patients with early-stage Parkinson’s disease exhibit greater densities of activated microglia in the midbrain and putamen, correlated with reduced DA transporter ligand activity. While chronic inflammation in Parkinson’s disease is well-recognized, the precise mechanism of neuroinflammation remains unclear. When α-synuclein accesses cells via the Toll-like receptor (TLR)-2, it can trigger a proinflammatory response as a damage-associated molecular pattern (DAMP) [154,155,156,157,158,159,160,161,162,163,164,165,166,167,168,169]. DAMPs, IL-1α, or mitochondrial ROS are also released by dying or injured cells, and when they engage with pattern recognition receptors (PRRs), they set off an innate immune response. Subsequent NLRP3 activation then increases the manufacturing of IL1β, which starts more innate immune reactions. Consequently, PRR-mediated reactions to DAMPs may be the cause of microglial activation in PD. Microglia progressively repolarized from an anti-inflammatory M2 to a proinflammatory M1 phenotype in animal models of neurodegeneration caused by 6-OHDA. Following repolarization, NF-κB triggers the synthesis of cytokines in M1 cells, which results in the transcription of procaspase-1 and interleukin. Together with caspase-1, these mechanisms generate the inflammasome NLRP3, which triggers the release of proinflammatory IL-1β; other proinflammatory proteins, like as TNFα and iNOS, that are generated by M1 cells also have a role in neurodegeneration in Parkinson’s disease. Lastly, TLR-4 contributes to neuroinflammation, as Noelker et al. found that TLR-4 deletion reduced the quantity of activated microglial cells and protected against SN dopaminergic degeneration [170,171,172,173,174,175,176,177,178,179,180]. Neuroinflammation with Parkinson’s disease is also influenced by adaptive immune responses. Numerous studies suggest that T-cell subpopulations play a role in the pathogenesis of Parkinson’s disease. For instance, patients with PD had considerable infiltration of both CD4 and CD8 T cells into their SN, with CD8 T-cell numbers being especially high. CD8 T cells appear to be crucial at the onset of the disease because this infiltration happens in early-stage Parkinson’s disease and decreases as the condition worsens. The population and activity changes of CD4 T-cells in PD patients, as well as an increase in human leukocyte antigen-DR positive antigen-presenting microglia, provide additional evidence of their role in neurodegeneration. This theory has been supported by research on Th17 cells’ function in Parkinson’s disease. Neurons eventually undergo NF-κB-dependent cell death and seem to be more vulnerable to IL-17 or autologous Th17 cells. Furthermore, pharmacological inhibition or CD4 T cell deletion reduces the expression of major histocompatibility complex (MHC) II in CNS myeloid cells and guards against the loss of tyrosine hydroxylase (TH) neurons in the ipsilateral SN pars compacta (SNpc) [177,178,179,180,181].

### 4.6. Role of Gut Dysbiosis in PD Pathology

**Figure 4:**
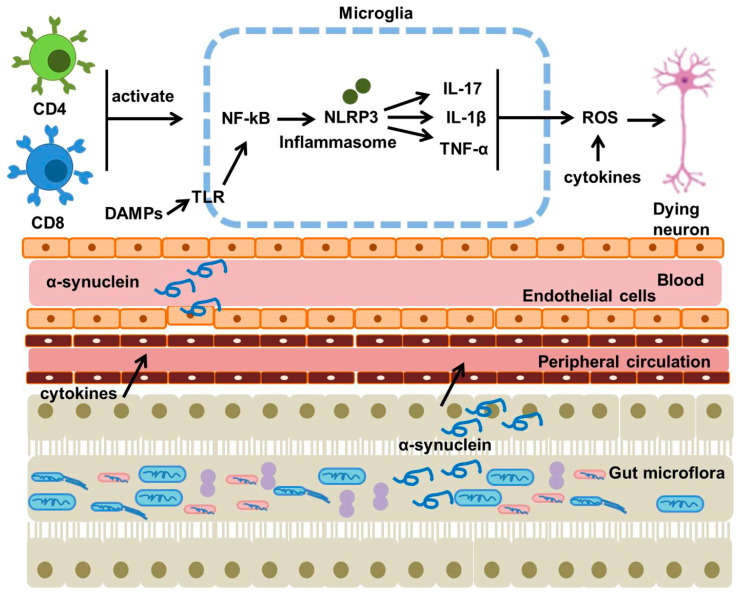
DAMPs (such as α-synuclein) initiate an innate immune response when they interact with pattern recognition receptors found in microglial cells. Activation of microglia subsequently raises levels of NF-κB and NLRP3, resulting in the upregulation of cytokines. Gut dysbiosis communicates with the CNS and enteric nervous system through metabolites, hormones, and the immune system, thereby facilitating neuroinflammation. This figure was generated by using Microsoft PowerPoint. This figure was modified from Xu Dong-Chen et al., 2023 [103].

There is much interest in the connection between gut microbiota and neurological disorders. The central neural system, enteric nervous system, autonomic nervous system, and hypothalamic–pituitary–adrenal axis are all included in gut–brain microbiota signaling. Metabolites, hormones, the immune system, and afferent nerves are all involved in the signaling pathways that connect the central nervous system to the enteric nervous system. Enteric nerve system inflammation can be mediated by microbiota (Figure 4).

Given that patients have elevated levels of calprotectin, a marker of intestinal inflammation, as well as zonulin and alpha-1-antitrypsin, indicators of intestinal barrier disruption, intestinal inflammation plays a role in the pathophysiology of Parkinson’s disease [182,183,184]. Systemic inflammatory reactions have been intimately linked to certain microbial species. For example, the abundance of Bacteroides was linked to plasma TNF levels, whereas the presence of Verrucomicrobiaceae was linked to plasma interferon (IFN)-γ levels. Roseburia promoted immunological homeostasis by negatively regulating the NF-κB pathway and upregulating innate immunity genes. The etiology of Parkinson’s disease may be influenced by gut bacteria and their metabolites through these effects on intestinal inflammation. In this context, lipopolysaccharide (LPS) is a noteworthy metabolite that increases intestinal permeability and α-synuclein accumulation in the enteric nervous system. Additionally, LPS treatment dramatically reduced intestinal epithelial cells’ levels of two tight junction proteins (zona occludens 1 and E-cadherin) in Thy1-α-synuclein animals, suggesting a connection between gut microbiota and Parkinson’s disease etiology [185,186,187,188]. The discovery that exposure to bacteria that produce curli enhances the deposition and aggregation of α-synuclein in intestinal ganglion cells and the brain, causing inflammation, is one example of evidence that microbiota plays a role in Parkinson’s disease. Injecting α-synuclein into the intestinal wall results in pathological alterations in the central nervous system, and numerous animal studies have also shown that α-synuclein disease can travel along the gut–brain axis from the intestine to the brain. Nevertheless, pathogenic processes may not always follow the gut–brain or brain–gut axes. As a disease progresses, pathologies may arise independently in the central nervous system and the enteric nervous system. Given this scenario, Arotcarena et al. suggested a mechanism in which endogenous α-synuclein is transmitted long-distance and in both directions via the general circulation between the intestinal tract and CNS [188,189,190,191,192,193,194]. Table 1 has shown the main molecular mechanisms involved in the pathophysiology of Parkinson disease.

## 5. Research Models of PD

Many models have enhanced our comprehension of the pathophysiology, etiology, and molecular mechanisms underlying Parkinson’s disease. The SNpc relies on SNpc dopaminergic neurons that can quickly deteriorate due to toxins such as 6-OHDA, MPP+, and MPTP, resulting in significant and unique motor impairments. The traditional models used in PD research are toxin-based models. Animal models for PD utilize both vertebrate and invertebrate species. *C. elegans* that overexpress α-synuclein exhibit damage to dopaminergic neurons, despite having few α-synuclein inclusions, and this degeneration is not progressive. Drosophila that overexpressed wild-type (WT), A53T, and A30P α-synuclein exhibited various Parkinson’s disease (PD) characteristics, including Lewy body-like filamentous inclusions and age-related, selective loss of dopaminergic neurons [195,196,197,198,199,200]. However, they lack the intricacy of vertebrates in expressing α-synuclein, and these models are unable to display important clinical characteristics such as stiffness, bradykinesia, and resting tremors. Although transgenic PD mice’s nigrostriatal system shows functional problems, their dopaminergic neurons do not show overt degenerative degeneration. The mouse prion promoter A53T α-synuclein transgenic mice (MitoPark) were the only animal models that replicated the entire spectrum of α-synuclein pathology seen in humans. As PD models, these MitoPark mice are therefore especially promising. They are produced when dopaminergic neurons’ gene encoding mitochondrial transcription-factor A (Tfam) is selectively disrupted. The mutation reduces the number of copies of mitochondrial DNA, which is comparable to traits seen in Parkinson’s disease in humans [201,202,203,204,205,206]. Furthermore, a respiratory chain shortage brought on by Tfam alteration leads to a gradual degenerative phenotype. Human Parkinson’s disease has both of these mitochondrial dysfunctional characteristics, highlighting the model’s value once more. A new model that highlights the aggregated, misfolded forms of α-synuclein found in Lewy bodies has recently become an important tool in the study of Parkinson’s disease. Researchers created preformed fibrils (PFFs) by incubating recombinant α-synuclein monomeric proteins under specific conditions. These fibrils mirror the structural elements of Lewy bodies and Lewy neurites. Synaptic dysfunction, alterations in cell excitability, and cell death can be caused by PFFs in primary neuronal cultures from WT mice and cell lines that overexpress disease-related proteins. Intracerebral injection of PFFs into the dorsal striatum results in behavioral deficits, neurodegeneration in the SNpc, and dysregulation of striatal DA release in mice overexpressing disease-related proteins or non-transgenic animals [207,208,209,210,211,212]. With early α-synuclein pathology in PD-relevant brain regions and the onset of DA dysfunction, nigral degeneration, and motor deficits months after induction, this type of model shows a longer time course of degeneration than other models, indicating a progression that is comparable to that of the human condition. The model’s failure to consistently produce pathogenic PFFs has limited the repeatability of the results and the investment in a model with a disease time course that takes several months to create.

## 6. Therapeutic Strategies for PD

### 6.1. Commercially Available Drugs for PD

Numerous medications have become effective therapies; we distinguish between them based on their pharmacological goals and list them in Table 2.

#### 6.1.1. Levodopa

Levodopa (L-DOPA), a traditional treatment for Parkinson’s disease, includes a number of unfavourable side effects, such as drug-induced dyskinesias and motor-response oscillations. These motor problems finally result from non-physiological pulsatile striatal DA receptor stimulation and are mediated by both presynaptic and postsynaptic pathways. Due to the short half-life of L-DOPA, as well as variations in gastrointestinal absorption and blood-brain barrier transit, discontinuous drug delivery is the primary source of maladaptive neuronal responses. Novel sustained-release formulations of L-DOPA and continuous delivery methods are constantly being developed to solve these issues. These include subcutaneous distribution using mini pumps and intestine delivery using percutaneous endoscopic gastro jejunostomy tubes [213,214,215,216,217].

#### 6.1.2. DA Agonists

Two kinds of DA receptors are found in spiny neurons in the striatal medium. Dopaminomimetics, like the ergot alkaloid bromocriptine, are receptor agonists that specifically target the D2 receptor family. In addition to activating 5-hydroxytryptamine (5-HT) receptors, including the 5-HT2B subtype, ergot alkaloids are ergoline derivatives. They have, however, been linked to pleuropulmonary fibrosis and cardiac valvular fibrosis, which raises serious safety concerns. Non-ergoline medications, on the other hand, are favored for treating Parkinson’s disease because they do not have this problem. DA agonists are excellent options for adjunct therapy in individuals with motor fluctuations since they have a longer half-life than L-DOPA. They are less effective overall than L-DOPA, though, and they are more likely to impair impulse control and induce drowsiness. Among DA agonists, apomorphine is distinct in that it acts on both D1 and D2 receptors simultaneously and has an identical affinity for L-DOPA. Limiting motor-response variability and reducing pre-existing L-DOPA-induced dyskinesias have been associated with continuous subcutaneous apomorphine infusions. New apomorphine formulations for sublingual usage are now being developed in clinical settings [217,218,219,220,221,222,223,224,225].

#### 6.1.3. Catechol-O-Methyltransferase and Monoamine Oxidase Type B Inhibitors

Catechol-O-methyltransferase (COMT) ortho-methylates L-DOPA through a secondary metabolic pathway during peripheral metabolism. The bioavailability and half-life of L-DOPA are enhanced when COMT is suppressed. Because of this impact, L-DOPA and COMT inhibitors are now used as part of the first-line treatment for PD patients. Entacapone and opicapone are two of the three COMT inhibitor formulations that are currently approved for clinical usage. In glial cells, monoamine oxidase type B (MAO-B) is the main mechanism for clearing synaptically produced dopamine. The action of DA is prolonged and its synaptic concentrations are raised when MAO-B is inhibited (for example, by the selective inhibitor selegiline). But while selegiline is irreversible, safinamide is a reversible MAO-B inhibitor that can be used to treat Parkinson’s disease [225,226,227,228,229,230].

#### 6.1.4. Non-Dopaminergic Targets

Even if dopaminergic medication has a great effect on PD symptoms, other target therapies are still required. First, L-DOPA-resistant (“non-dopaminergic”) motor features (e.g., treatment-resistant tremors, postural instability, frozen gait, swallowing difficulties, and speech disturbances), as well as L-DOPA-induced dyskinesia and motor fluctuations, require new treatments. Amantadine is now the only readily available and efficient pharmaceutical treatment for L-DOPA-induced dyskinesia; it is thought to be an antagonist of the N-methyl-D-aspartate receptor. Second, new therapies need to target PD’s non-motor symptoms, namely, autonomic failure, cognitive impairment, and depression. The fact that many non-motor symptoms do not improve with DA replacement therapy, and that some are even triggered or made worse by it, is a significant issue. Cholinesterase inhibitors help people with dementia and Parkinson’s disease who have cognitive impairments. This favourable result may be linked to dementia’s substantial loss of cholinergic projections from the nucleus basalis of Meynert [218,231,232]. The best treatment for psychotic symptoms in PD is clozapine. Lastly, autonomic dysfunction is rather prevalent in Parkinson’s disease, particularly in its latter stages. There are several pharmacological treatments that primarily target the autonomic nerve system. Mineralocorticoid fludro cortisone, adrenergic agents (like midodrine and etilefrine), anti-muscarinics (like tolterodine, oxybutynin, or trospium chloride) for urinary urgency or incontinence, noradrenaline precursor (droxidopa) for orthostatic hypotension, and prokinetic medications (like macrogol or lubiprostone) for constipation are some of these [218,219,220,221,232,233].

### 6.2. Drugs for PD Treatment Under Clinical Trials

The newly discovered medications are being tested in a number of clinical trials (Table 3). A few of them have displayed signs of becoming PD treatment candidates. Tavapadon is a strong, very selective partial agonist for the DA D1/D5 receptor that is taken orally. In a clinical experiment (NCT02847650), tavapadon demonstrated a greater improved impact than a placebo, which served as the foundation for the present clinical phase III trial (NCT04760769). IRL790 was created as an experimental treatment for PD’s L-DOPA-induced dyskinesia, impulse control problem, and psychosis because it may interact with the DA D3 receptor. In the study, patients with severe Parkinson’s disease on IRL790 reported fewer motor symptoms and no significant side effects. Phase II clinical trials are continuing in the early stages of follow-up stages (NCT03368170). Deferiprone decreased SN iron deposition and the advancement of motor impairment in PD patients in Phase II randomized double-blind placebo-controlled clinical studies (NCT00943748, NCT01539837). In a Phase I dose-escalation study in early PD patients (NCT03204929), Cu (II) ATSM had a beneficial effect by avoiding lipid peroxidation, which raises the possibility of PD treatment. A monoclonal antibody called prasinezumab targets α-synuclein. Its discovery has drawn much attention as a potential treatment approach against a crucial target in Parkinson’s disease (PD), but in a recent phase II clinical research (NCT03100149), it had no therapeutic benefit when compared to a placebo and raised safety concerns [234,235,236,237,238,239,240]. However, additional research may be necessary to confirm its effectiveness, and fresh clinical trials are currently being conducted (NCT04777331). In the double-blind randomized clinical phase I trial (NCT04056689) conducted by Denali Therapeutics, DNL151, an LRRK2 inhibitor, showed a fairly clear therapeutic benefit on Parkinson’s disease. Since then, it has started clinical phase II (NCT05348785) and III phase studies (NCT05418673). If the outcomes are positive, it could demonstrate how effective LRRK2 is at treating Parkinson’s disease.

### 6.3. Phytochemical-Based Therapeutic Strategy for PD Treatment

New remedies ought to be created in light of the negative impacts of Western medicine and the intrusiveness of external physical interventions. The usefulness of several natural compounds derived from medicinal plants and their formulations in the treatment of Parkinson’s disease has been the subject of numerous studies in recent years. Several natural items have been shown to regulate Parkinson’s disease at the molecular level. The list of compounds suggested to have therapeutic potential for PD treatment are summarized in Table 4.

#### 6.3.1. Phenol

Yang et al. showed the preventive benefits of curcumin on the damaged hippocampus using a 6-OHDA-induced PD model. Significant improvements in mental status, elevations of dopamine and norepinephrine, hippocampus tissue regeneration, and activation of proteins involved in cell survival-related pathways, including BDNF, tropomyosin receptor kinase (Trk) B, and phosphoinositide 3-kinase (PI3K), were among these advantages. This finding is supported by additional research using 6-OHDA PD models, which demonstrate that curcumin promotes neural regeneration via activating Trk/PI3K signalling, which reduces TNF-α and caspase activity while raising BDNF levels. Additional investigation into curcumin’s mode of action suggests that it requires at least some interaction with a nicotinic acetylcholine receptor (α7).

Curcumin reduces aberrant turning behavior and increases the survival of striatal TH fibers and neurons in the SNpc by this mechanism. Additionally, curcumin inhibits a variety of inflammatory substances, such as glial fibrillary acidic protein, cytokines, ILs, chemokines, inflammatory enzymes, cycloxygenase-2, and cyclin D1. Moreover, curcumin inhibits many players in apoptotic pathways, including c-Jun Nterminal kinase (JNK), iNOS, LPS-induced TNF-α, IL-1β, and IL-6 [240,241,242,243,244,245,246]. Data demonstrating that curcumin affects the impact of numerous inflammatory mediators further supports these anti-inflammatory qualities. Resveratrol has been demonstrated to have neuroprotective benefits in PD models derived from 6-OHDA, MPP+, and rotenone in both in vitro and in vivo experiments. Resveratrol blocks apoptosis by activating the pro-survival PI3K/protein kinase B (Akt) pathway, increasing the ratio of B-cell lymphoma (Bcl-2) to Bcl-2-associated X (Bax), and decreasing cytochrome C release, which keeps caspase-3 dormant. Additionally, following exposure to MPTP/MPP+ and rotenone, resveratrol boosts antioxidant defenses and reduces the generation of ROS. Resveratrol prevents mitochondrial dysfunction in experimentally produced Parkinson’s disease (PD) by boosting complex-I activity and mitochondrial biogenesis while reversing alterations in mitochondrial shape and membrane potential. Resveratrol reduces α-synuclein expression in the striatum and promotes autophagic clearance of α-synuclein following sirtuin (SIRT) 1 activation in a number of animal models. Similarly to gastrodin activity, resveratrol also increases autophagic flow by activating the HO-1 and mitogen-activated protein kinase (MAPK) pathways. Neuroprotection also involves the control of astroglial activity. When taken with L-DOPA, resveratrol exhibits synergistic effects, which is encouraging for therapeutic use [247,248,249,250,251,252,253,254,255,256,257,258,259].

#### 6.3.2. Alkaloids

Berberine treatment dramatically reduced dopaminergic neuronal degeneration in the SN compacta and improved motor impairment in mice treated with MPTP. Additionally, berberine increased autophagy linked to microtubule-associated protein light chain 3 (LC3-II) and lowered α-synuclein levels. Additionally, berberine activated adenosine 5′-monophosphate (AMP)-activated protein kinase (AMPK). One noteworthy advantage of AMPK is that it protects cells against rotenone and reduces α-synuclein-induced toxicity. Another study examining berberine’s effects in animal models revealed that mice given berberine had dramatically reduced levels of NLRP3 inflammasome and NLRP3-associated neuroinflammation [260,261,262,263]. The phenylalanine–tyrosine–DA pathway’s rate-limiting enzyme, TH, may be the target of berberine’s particular mode of action. This route uses tetrahydrobiopterin as a coenzyme to produce L-DOPA and supply DA to the brain. It has been demonstrated that bacterial nitroreductase converts berberine to dihydroberberine in the intestines. Tetrahydrobiopterin concentrations rise as a result of this process, which also provides and boosts TH activity. In the end, the creation of L-DOPA by gut bacteria is accelerated. Isorhynchophylline (IRN) facilitates the removal of WT, A53T, and A30P α-synuclein aggregates from neuronal cells via the autophagy–lysosome route. IRN-induced autophagy depends on Beclin 1 function but is not mediated by the mTOR pathway. IRN treatment significantly decreased endoplasmic-reticulum stress responses mediated by MPP+. Furthermore, it appears that IRN suppression of the apoptotic signal-regulating kinase 1 (ASK1)/JNK pathway inhibits mitochondria-dependent apoptosis, indicating neuronal protection [258,259,260,261,262,263,264,265,266,267,268,269,270,271].

#### 6.3.3. Flavonoids

Nrf2 is necessary for the anti-parkinsonian actions of the flavonoid puerarin. Puerarin controlled the phosphorylation of Fyn and GSK-3β in the ventral midbrain of mice given MPTP. De novo glutathione synthesis is facilitated by Nrf2 increase in the nucleus, which is facilitated by the Fyn/GSK-3β pathway. The information that is now available suggests that puerarin uses progesterone receptors to enhance dopaminergic neuron survival, proliferation, and differentiation. Additionally, puerarin inhibits GSK-3 activity in neurons and reduces inflammatory responses by acting on the PI3K/Akt pathway, which limits the generation of caspase-3 and the apoptosis that goes along with it. The way puerarin prevents MPP+-induced neuroblastoma SH-SY5Y cell death is explained by these interactions, as well as the regulation of nuclear p53 accumulation. The injection of baicalein restored the proinflammatory cytokine increase, dopaminergic neuronal loss, and motor impairment caused by MPTP. Furthermore, baicalein prevented disease-associated proinflammatory microglia from activating and proliferating. This protective effect’s fundamental mechanism is most likely the suppression of the NLRP3/caspase-1/gasdermin D pathway. Pyroptosis, a form of programmed cell death linked to this system, contributes to the degeneration of dopaminergic neurons. NLRP3 inflammasome activation initiates pyroptosis, which, in turn, stimulates caspase-1 maturation [271,272,273,274,275,276,277,278,279,280,281,282,283]. The pyroptosis executive protein gasdermin D is then oligomerized by caspase-1, which promotes the release of proinflammatory IL-1β and IL-18. Additionally, studies have shown that baicalein protects synaptic plasticity and decreases α-synuclein aggregation via acting on the BDNF/TrkB/Cyclic AMP response-element binding protein (CREB) pathway. Baicalein’s medicinal benefits have been further demonstrated by numerous studies employing it in various settings. For example, patients’ gait function was greatly restored when baicalein and low-dose L-DOPA were combined, reaching a level similar to that of high-dose L-DOPA treatment, despite some L-DOPA side effects. Lastly, baicalein decreased Bcl-2, phosphorylated extracellular signal-regulated kinases, increased Bax and cleaved caspase-3, and inhibited rotenone-induced ROS overproduction (ERK) [284,285,286,287].

#### 6.3.4. Terpenoids

Celastrol alleviates nigrostriatal dopaminergic degeneration and motor impairments by inhibiting the NLRP3 inflammasome via the Nrf2-NLRP3-caspase-1 pathway. Celastrol stimulates mitophagy, autophagy, and autophagosome biogenesis in neurons, most likely in connection with MAPK pathways. Celastrol prevents ATP loss, mitochondrial membrane depolarization, and dopaminergic neuronal death in MPP+-induced PD cell models. Additionally, studies employing these models indicate that celastrol preserves mitochondrial quality by enclosing faulty mitochondria in autophagosomes for eventual destruction. Following fibril-induced microglial activation mediated by α-synuclein, triptolide therapy inhibited microglial activation and reduced the release of proinflammatory cytokines. The medication specifically inhibited NF-κB’s activity in the inositol polyphosphate 5-phosphatase (SHIP) 1 pathway by targeting the miR155-5p/Src homology 2 (SH2) domain. Research indicates that the overexpression of miR155-5p triggers NF-κB activity by suppressing SHIP1. Triptolide reduces the inflammatory response via interfering with the regulation of SHIP1 by miR155-5p. Triptolide’s anti-inflammatory effects were shown to be lessened when metabotropic glutamate receptor subtype 5 (mGlu5) was blocked in an LPS-induced Parkinson’s disease model. Furthermore, the impact of triptolide on microglia-induced astrocyte activation in vitro and in vivo seems to be mediated by mGlu5. Additionally, triptolide has been reported to be a strong inducer of autophagy in neuronal cells, assisting in the removal of different types of α-synuclein through the autophagic pathway [288,289,290,291,292,293].

#### 6.3.5. Saponins

Promoting hippocampus CA3 α-synuclein expression, restoring glutamate in the CA3-schaffer collateral-CA1 route, and gradually raising postsynaptic density-95 expression are the neuroprotective mechanisms behind ginsenoside Rb1-improvements to synaptic plasticity. Ginsenoside Rb1 therapy significantly reduced apomorphine-induced rotations, SN inflammation, and DA (plus metabolites) depletion in the striatum of rats given LPS. These effects could be linked to the NF-κB signaling pathway being inhibited. The ginsenoside Rg3 decreased ROS levels in the SN while increasing the number of TH-positive neurons in the SN, the mean density of TH-positive nerve fibers, and the DA concentration in the striatum [294,295,296,297].

## 7. Discussion and Perspectives

The key pathological characteristics of Parkinson’s disease (PD) involve the degeneration of dopaminergic neurons in the SN and the accumulation of α-synuclein. PD results from various complex processes, such as ferroptosis, neuroinflammation, mitochondrial dysfunction, gut dysbiosis, oxidative stress, α-synuclein aggregation, and additional factors. Interactions play a crucial part in the progression and development of Parkinson’s disease. Besides the toxin, transgenic, and PFFs models previously discussed, additional models are essential for experimental studies to propel PD research forward, as the complex mechanisms driving the effects of these strategies on PD still need exploration. The main drug employed in managing Parkinson’s disease (PD) remains L-DOPA, while additional medications like COMT inhibitors and MAO-B inhibitors are often utilized alongside L-DOPA. Additionally, individuals with advanced Parkinson’s disease often do not adequately respond to existing medications, which greatly diminishes their quality of life. Consequently, additional drugs are still necessary for therapy. Researching new drugs that focus on ferroptosis, oxidative stress, α-synuclein aggregation, and other related factors can be founded on the mechanisms underlying Parkinson’s disease. The associated medicines currently under development have not shown favorable outcomes and might still require further investigation. Alongside well-known chemical, biological, and various other treatments for Parkinson’s disease, several studies have verified the efficacy of traditional Chinese medicines, which show significant potential for managing the condition. Traditional Chinese medicines contain substances that have complex pharmacological properties and cure the degenerative pathways of Parkinson’s disease (PD), including OS, neuroinflammation, and α-synuclein aggregation. Many natural medications, including tanshinone and andrographolide, which have been documented to have anti-inflammatory properties and may be useful in the treatment of Parkinson’s disease, have been suggested for additional research in the Zhu et al. study [298]. Furthermore, given the intricate mechanisms, multi-drug combinations, such as a mix of chemical and biological medications or natural small molecules, may provide a fresh viewpoint on the treatment of Parkinson’s disease. Some surgical treatment approaches additionally contain patients’ new ideal strategies in addition to medication therapies. High-frequency (100–200 Hz) electrodes can create deep brain interference, which mimics the effects of a lesion without causing damage to brain tissue [220]. Utilizing this method, a clinical experiment has improved gait-function recovery in PD patients by combining treadmill gait training with transcranial direct current stimulation (NCT04591236). Recent research has also shown that transcutaneous magnetic stimulation of the spinal cord, which can non-invasively stimulate neural components, may be a viable treatment option for gait abnormalities (NCT05008289). The application of stem cells is another exciting field of study. Autologous iPSCs were used in a clinical trial to differentiate patient-derived midbrain dopaminergic progenitor cells. The putamen was subsequently transplanted with these cells. Following treatment, patients’ PD symptoms subsided, enabling a 6% reduction in the daily dose of L-DOPA equivalent [297]. These imply that scientists might find a new therapeutic strategy in the field of stem cell transplantation.

The review presented in this work covers a wide range of current data on the pathophysiology of PD. We have particularly focused on describing key molecular processes in PD, as well as the traditional research models, clinical diagnostic standards, documented medication therapeutic approaches, and recently disclosed drug candidates in clinical trials. By reviewing recent advances and highlighting future research directions, we aim to shed light on the promising role of drugs in addressing unmet therapeutic needs in Parkinson disease.

## Figures and Tables

**Table 1:** Major molecular and cellular mechanisms involved in Parkinson’s disease pathology.

Mechanism	Role in PD Pathology	Effects
α-Synuclein Aggregation	Misfolded α-synuclein forms toxic oligomers and fibrils, accumulating as Lewy bodies in dopaminergic neurons	- Disrupts synaptic function - Impairs protein degradation - Spreads prion-like
Oxidative Stress (OS)	Excess ROS from mitochondrial impairment, dopamine metabolism, and inflammation	- Lipid peroxidation - DNA/protein damage - α-Synuclein misfolding
Ferroptosis	Iron-dependent, lipid-peroxide-driven cell death pathway observed in dopaminergic neuron degeneration	- Increased iron accumulation - GPX4 depletion - Membrane lipid peroxidation
Mitochondrial Dysfunction	Impaired complex I activity in the electron transport chain leads to energy failure and ROS generation	- ATP depletion - Increased oxidative stress - Cytochrome c release and apoptosis
Neuroinflammation	Activation of microglia and astrocytes promotes chronic inflammation and release of pro-inflammatory cytokines	- TNF-α, IL-1β release - Increased BBB permeability - Further neuron damage
Gut Dysbiosis	Altered microbiota composition affects gut-brain axis, promoting inflammation and α-synuclein aggregation	- Increased intestinal permeability - Elevated endotoxins (LPS) - Immune activation

This table was modified from Xu Dong-Chen et al., 2023 [103].

**Table 2:** List of commercially available drugs for PD treatment.

Class	Drug	Therapeutic Applications
L-DOPA preparation	L-DOPA/benserazide tablet Carbidopa/L-DOPA tablet Carbidopa/L-DOPA controlled-release tablet	Parkinson’s syndrome Parkinson’s syndrome Parkinson’s syndrome, wearing-off, dyskinesia
DA agonists	Pramipexole tablet Ropinirole tablet Piribedil Transdermal rotigotine Injected apomorphine	Parkinson’s early syndrome, L-DOPA adjunct, wearing-off, dyskinesia Parkinson’s early syndrome, L-DOPA adjunct, wearing-off, dyskinesia Tremor, DA adjunct Parkinson’s early syndrome, L-DOPA adjunct, wearing-off, dyskinesia Wearing-off, L-DOPA-induced dyskinesias
N-methyl-D-aspartate receptor antagonist	Amantadine	Parkinson’s early syndrome, L-DOPA adjunct
Adenosine A2a receptor antagonists	Istradefylline	Wearing-off
Others	Clozapine	Dyskinesia
Anticholinergics	Benztropine Trihexyphenidy	Parkinson’s early syndrome, L-DOPA adjunct Parkinson’s early syndrome, L-DOPA adjunct
COMT inhibitors	Entacapone Opicapone Tolcapone	Wearing-off, dyskinesia Wearing-off, dyskinesia Wearing-off
MAO-B inhibitors	Selegiline Rasagiline Safinamide Zonisamide	Parkinson’s early syndrome, wearing-off, dyskinesia Parkinson’s early syndrome, L-DOPA adjunct, Wearing-off, Dyskinesia Wearing-off, Dyskinesia Wearing-off

This table was modified from Xu Dong-Chen et al., 2023 [103].

**Table 3:** List of drugs for PD treatment under clinical trials.

Therapeutic Strategy	Name	Target and Classification
DA receptor agonists	PF-06649751/CVL 751/Tavapadon PF-06669571 PF-06412562 KDT-3594/AM-006 Lu-AF28996	Small molecular DA D1/D5 agonist Small molecular DA D1 agonist Small molecular DA D1 agonist Small molecular DA agonist Small molecular DA D1/D2 agonist
Anti-α-synuclein aggregation therapy	Prasinezumab/PRX002/RO7046015 MEDI-1341/TAK-341 Lu AF82422 UCB7853 UCB 0599 Kenterin/Enterin-01 Ambroxol	Monoclonal antibody Monoclonal antibody Monoclonal antibody Monoclonal antibody Small molecular SNCA antagonists Small molecular SNCA antagonist Small molecular decrease in the cerebrospinal fluid α-synuclein level
Targeting ferroptosis	Cu(II)ATSM	Small molecular Peroxynitrite scavenger
Serotonin receptor agonists or antagonists	Landipirdine/SYN120/RO-5025181 SEP-363856	Small molecular dual 5-HT6/5-HT2 antagonist Small molecular 5-HT1A agonist
Others	CNM-Au8 NLY01/NLY01-AD	Small molecular Small molecular GLP1R agonist
Gene therapy	AAV2-GDNF LY3884961/PR001A	AAV2-GDNF delivered to the putamen Glucocerebrosidase gene therapy by intra cisterna magna administration
Cell-based therapy	NTCELL ISC-hpNSC ANGE-S003	Immunoprotected (alginate-encapsulated) porcine choroid pplexus cells Neural stem cells Neural stem cell
Botanical-based medication	DA 9805 Hypoestoxide WIN-1001X	Natural compounds Plant-based herbal dry powder Plant-based herbal extract
Kinase inhibitors	SUN-K706/Vodobatinib/SCC138/K0706 Nilotinib/Tasigna/AMN-107 Radotinib Dihydrochloride/IY-5511 BIIB-122/DNL151 DNL-201	Small molecular Bcr-Abl antagonist Small molecular Bcr-Abl antagonist Small molecular Bcr-Abl antagonist Small molecular LRRK2 antagonist Small molecular LRRK2 antagonist
Muscarinic and nicotinic acetylcholine receptor agonists	Blarcamesine/AF710B/ANAVEX 2-73	Small molecular Muscarinic acetylcholine receptor M1 agonist
Acetylcholinesterase antagonists	Buntanetap/ANVS-401	Small molecular AchE antagonist/TAU antagonist
Adenosine A2a receptor antagonists	KW-6356 Caffeine	Small molecular adenosine A2A receptor antagonist Small molecular selective adenosine A2A antagonist
N-methyl-D-aspartate receptor (NMDAR) modulators	NBTX 001 NYX-458 DAAOI-P	Small molecular NMDAR modulato Small molecular NMDAR modulator Small molecular D-amino acid oxidase inhibitor

This table was modified from Xu Dong-Chen et al., 2023 [103].

**Table 4:** List of natural compounds with therapeutic potential for PD treatment.

Class	Name	Targets
Phenol	Curcumin Resveratro	Trk/PI3K, JNK PI3K/Akt, SIRT 1, MAPK
Saponin	Ginsenoside Rb1 Ginsenoside Rg3	NF-κB NF-κB
Terpenoid	Celastrol	MAPK, Nrf2-NLRP3-caspase-1
Flavonoid	Puerarin Baicalein	Fyn/GSK-3β, PI3K/Akt NLRP3/caspase-1/gasdermin D, BDNF/TrkB/CREB
Alkaloid	Berberine Isorhynchophylline	AMPK ASK1/JNK

This table was modified from Xu Dong-Chen et al., 2023 [103].

## Data Availability

The original contributions presented in the study are included in the article; further inquiries can be directed to the corresponding author.
